# Effects of medium chain triglycerides with organic acids on growth performance, fecal score, blood profiles, intestinal morphology, and nutrient digestibility in weaning pigs

**DOI:** 10.5713/ab.21.0469

**Published:** 2022-03-01

**Authors:** Tae Wook Goh, Jinsu Hong, Dong Hyun You, Yeong Geol Han, Seung Ok Nam, Yoo Yong Kim

**Affiliations:** 1 Department of Agricultural Biotechnology, and Research Institute of Agriculture and Life Sciences, Seoul National University, Seoul 08826, Korea; 2 Department of Animal Science, South Dakota State University, Brookings, SD 57006, USA

**Keywords:** Growth Performance, Intestinal Morphology, Medium Chain Triglycerides, Organic Acid, Weaning Pig

## Abstract

**Objective:**

This study was conducted to evaluate the effects of medium chain triglycerides (MCT) with organic acids (OA) on growth performance, fecal score, blood profiles, intestinal morphology, and nutrient digestibility in weaning pigs.

**Methods:**

A total of 120 weaning pigs ([Yorkshire×Landrace]×Duroc) with an average body weight (BW) of 8.00±0.87 kg were assigned in five treatments considering sex and initial BW in 3 replications with 8 pigs per pen in a randomized complete block design. The experimental diets included a corn-soybean meal based basal diet with or without 0.1% or 0.2% MCT and 0.1% OA. The pigs were fed the diets for 5 weeks (phase 1, 0 to 2 weeks; phase 2, 3 to 5 weeks). A total of 15 barrows with an average BW of 12.48±0.37 kg were used to evaluate the nutrient digestibility by total collection method. The BW and feed intake were measured at the end of each phase. Blood samples and small intestine samples were collected at the end of each phase, too.

**Results:**

Supplementing 0.1% MCT with 0.1% OA showed greater BW for week 5 and average daily gain (ADG) for overall period than control diet. Supplementing 0.1% MCT increased (p<0.05) ADG and improved (p<0.05) gain:feed ratio for phase 1. Dietary MCT and OA did not affect the fecal score and blood concentration of cortisol, immunoglobulin G, tumor necrosis factor-α, interleukin-1β (IL-1β), IL-6, and IL-10 in weaning pigs. Pigs fed the diets with 0.1% MCT had greater (p<0.05) villus height of duodenum and ileum for phase 1. Also, pigs fed the diet with 0.1% OA showed greater (p<0.05) villus height and villus height to crypt depth ratio of duodenum for phase 2. There was no significant difference in nutrient digestibility and nitrogen retention of pigs.

**Conclusion:**

Addition of 0.1% MCT with 0.1% OA in weaning pig’s diet improved growth performance partly by enhancing intestinal morphology in weaning pigs.

## INTRODUCTION

Weaning pigs experience highly stressful events when they are weaned from the sow [1]. To alleviate the detrimental effects of weaning, antibiotic growth promoters (AGPs) have been widely used in weaning pigs’ diet. However, the use of AGPs in animal feed have been forbidden due to the antibiotic resistance problem [2]. Several feed additives as alternatives to AGPs have been proposed to overcome the increased mortality due to the ban on antibiotic use in animal feed [2]. Among the alternatives, medium chain triglycerides (MCT) and organic acids (OA) have been widely researched and applied in weanling pig diets as promising alternatives to AGPs [3].

Medium chain triglycerides have nutritional and metabolic effects including speeding up digestion and improving passive absorption and effects on oxidation status. Therefore, they are particularly useful for the improving nutritional ingestion in piglets [4]. It has been reported in many previous studies that MCT has a positive effect on improving the growth performance of piglets, especially during the second weeks after weaning [5]. In addition, MCT has antimicrobial effects on gram-positive cocci [6] and *Escherichia coli* (*E. coli*) [7] and protective effects on the villi [5].

Organic acids can lower gastric pH which is essential for the proper digestion of nutrients and in particular protein digestion for improving growth performance and prevention of infection by diarrhea-causing micro-organisms [8]. Organic acid and their salts, especially formic acid, and potassium formate, have the advantage of improving the feed intake and growth rate of piglets [8]. They also affect the gastrointestinal environment and the digestive process [9]. Moreover, they have effects on intracellular acidification, and possibly act to inhibit bacteria [9].

Although both MCT and OA have beneficial effects for weaning pigs, there are few studies on supplementing MCT and OA in weaning pigs’ diet at the same time. Kuang et al [3] investigated the effects of combinations of MCT and OA growth performance in weaning pigs. Animals supplemented with 0.3% MCT and OA showed higher feed intake (p<0.01) during the first two experimental weeks and had a higher body weight (BW) (p<0.05) at the end of each of the four experimental weeks than the control. Lei et al [10] also conducted the experiment to compare the effects of MCT and OA on growth performance in weaning pigs. Weaning pigs fed the combination of 0.2% and 0.4% MCT and OA had higher (p<0.05) BW than those of control groups on day 7, 14, and 21 of the experiment.

However, there is insufficient evidence to verify the synergic effect of MCT and OA on growth performance, gastrointestinal environment, and nutrient absorption in weaning pigs. Thus, it was hypothesized that synergic effects of MCT and OA could improve gastrointestinal environment and nutrient absorption, leading to an improvement of growth performance in weaning pigs. Therefore, this study was conducted to evaluate the effects of MCT with OA on growth performance, fecal score, blood profiles, intestinal morphology, and nutrient digestibility in weaning pigs.

## MATERIALS AND METHODS

All experimental procedures involving animals were conducted in accordance with the Animal Experimental Guidelines provided by the Seoul National University Institutional Animal Care and Use Committee (SNUIACUC; SNU-170424-3-1).

### Experimental animals and management

A total of 120 weaning pigs ([Yorkshire×Landrace]×Duroc) with initial BW of 8.00±0.87 kg were allotted to one of five treatments considering sex and initial BW in 3 replicates with 8 pigs per pen in a randomized complete block design. Pigs were randomly allotted to their respective treatments by the experimental animal allotment program (EAAP) [11]. Pigs were housed in an environmentally controlled facility. The pens were fully concrete floored (1.54×1.96 m) and equipped with a feeder and, water nipple. The experimental period was 5 weeks (phase 1, 0 to 2 weeks; phase 2, 3 to 5 weeks).

### Experimental design and diet

Dietary treatments included: i) CON (corn-soybean meal (SBM) based diet), ii) LM (corn-SBM based diet + 0.1% MCT), iii) LMO (corn-SBM based diet + 0.1% MCT + 0.1% OA), iv) HM (corn-SBM based diet + 0.2% MCT), v) HMO (corn-SBM based diet + 0.2% MCT + 0.1% OA). In the present study, the MCT and OA products were provided by E&T company (E&T CO., Ltd, Daejeon, Korea). The MCT product contained 49.65% C6:0 and 43.01% C8:0 and the OA product contained 65% calcium formate, 15% citric acid, 10% fumaric acid. Experimental diets were formulated for the 2 phases, including weaning phase 1 and weaning phase 2. All nutrients in the experimental diets except crude protein (CP) and metabolizable energy (ME) met or exceeded the nutrient requirements of the National Research Council (NRC) [12]. The ME and CP were determined to meet NRC [13]. The formula and chemical composition of the experimental diet are presented in Table 1 and 2.

### Growth performance

Body weight and feed intake were measured at the end of each phase to calculate the average daily gain (ADG), average daily feed intake (ADFI), and gain:feed ratio (G:F ratio). In addition, feed given to all piglets was recorded each day, and feed waste in the feeder was recorded at the end of each phase.

### Fecal score

Observations of fecal score were made every 08:00 throughout the feeding trial (35 days). Data were recorded by one trained researcher for each pen. Fecal scores were given according to the condition of feces (0 = normal feces; 1 = moist feces; 2 = mild feces; 3 = watery diarrhea) [14]. The score of 2 or 3 was considered severe diarrhea. Slightly wet feces on the rump area were used to designate contaminated piglets. After recording data, we cleaned away the feces by wiping off the fecal areas or the pig’s butt preparing for a new measurement the next day.

### Blood collection and analysis

Blood samples were taken from the jugular vein of five pigs near the average BW in each treatment after 3 hours of fasting at initial day and the end of each phase for measuring cortisol, immunoglobulin G (IgG), tumor necrosis factor-α (TNF-α), interleukin-1β (IL-1β), IL-6, and IL-10. All blood samples were collected in the serum tubes (SST II Advance; BD Vacutainer, Becton Dickinson, Plymouth, UK) and centrifuged at 1,957×g and 4°C for 15 min (5810R; Eppendorf, Hamburg, centrifuge 5810R, Germany). The sera were carefully transferred to 1.5 mL microtubes (MCT-150-C; AXYGEN. INC, Glendale, AZ, USA) and stored at −20°C until further analysis. The enzyme-linked immunosorbent assay (ELISA) were used to measure the following parameters: cortisol concentration (Microplate Reader, VERSA Max, Swine Cortisol ELISA Kit; Molecular Devices, San Jose, CA, USA) to assess stress; IgG concentration (Microplate Reader, VERSA Max, Pig IgG ELISA Kit; Molecular Devices, USA) to determine immune status; TNF-α (Microplate Reader, VERSA Max, Quantikine Porcine TNF-a TNFSF2 Immunoassay; Molecular Devices, USA), IL-1β (Microplate Reader, VERSA Max, Quantikine Porcine IL-1β IL-1F2 Immunoassay; Molecular Devices, USA), and IL-6 (Microplate Reader, VERSA Max, Porcine IL-6 Immunoassay; Molecular Devices, USA) concentrations to assess the degree of inflammatory cytokine changes; and IL-10 concentration (Microplate Reader, VERSA Max, Quantikine Porcine IL-10 Immunoassay; Molecular Devices, USA) to investigate the degree of ant-inflammatory cytokine changes.

### Nutrient digestibility

A total of 15 crossbred barrows, averaging 12.48±0.37 kg BW were allotted to individual metabolic crates (40×80×90 cm) in a completely randomized design with 3 replicates to evaluate the nutrient digestibility and nitrogen retention. The total collection method was used to determine the apparent total tract digestibility of dry matter (DM), CP, crude ash, and crude fat [15]. After a 5-day adaptation period, there was a 5-day collection period. To determine the first and last day of collection, 8 g of ferric oxide and chromium oxide were added to the first and last experimental diets as selection markers. During the experimental period, all pigs were fed the phase 2 diets twice a day, which provided three times the maintenance energy [16], at 7:00 and 19:00, and water was provided *ad libitum*. Collection of feces was started when the ferric oxide appeared in the feces and kept until the appearance of chromium oxide in the feces. Urine samples were collected during collection period in plastic containers containing 50 mL of 4 N H_2_SO_4_ to prevent evaporation of nitrogen prior to nitrogen retention analysis. Fecal and urinary samples were stored at −20°C until the end of collection period and the feces were dried in a drying oven at 60°C for 72 h and then ground to 1 mm in a Wiley mill (CT 193 Cyclotec; FOSS, Höganäs, Sweden) for chemical analysis including moisture, CP, crude fat, and crude ash contents by the Association of Official Analytical Chemists (AOAC) methods [17].

### Intestinal morphology

Three piglets of near average BW were selected for each treatment and euthanized at the end of each phase to collect small intestinal tissues. After removal of the digestive system, 6 to 8 cm samples from the middle of each duodenum, jejunum, and ileum were cut out, cleaned, and stored in 10% neutral-buffered formalin solution until further morphological analysis. The samples were then cut into two parts for each segment for measurement of the cross section and length of the intestine surface and then processed by the standard paraffin method. Sections (2 to 3 cm) were stained with hematoxylin and eosin, and examined under a light microscope (Leica DM500 microscope with Leica DFC425; Leica Microsystems Inc., Morrisville, NC, USA) was used for image analysis. The villus height (VH) was measured from the tip to the villus crypt junction. The crypt depth (CD) was measured as the depth between adjacent villi.

### Chemical analysis

The diets and feces were ground by Wiley mill (CT 193 Cyclotec; FOSS, Sweden) and then analyzed for DM (procedure 967.03; [17]), ash (procedure 923.03; [17]), ether extract (procedure 920.39; [17]). The nitrogen content was analyzed by using the Kjeldahl procedure with Kjeltec (KjeltecTM 2200; Foss Tecator, Sweden) and calculating the CP content (nitrogen×6.25; procedure 981.10; [17]).

### Statistical analyses

Differences among all treatments were carried out by comparing means according to the least significant difference multiple range tests, using the general linear model protocol of SAS (SAS Inst. Inc., Cary, NC, USA). Each pen was used as the experimental unit for growth performance and fecal score, while individual pig was used as the experimental unit for blood profiles, intestinal morphology, and nutrient digestibility. The data were analyzed as a 2×2 factorial treatment arrangements to find out the main effects of MCT and OA, as well as interaction between MCT and OA. Also, the data were analyzed by analysis of variance to compare the effects of MCT and OA against control. Differences were declared significant at p<0.05, while 0.05≤p<0.10 was considered to indicate a trend in the data.

## RESULTS AND DISCUSSION

### Growth performance

The dietary effects of MCT with OA on growth performance are presented in Table 3. An interaction between dietary MCT and OA was observed in the ADG for 3 to 5 weeks (MCT× OA, p = 0.04) such that there was no 0.1% OA effect with 0.1% MCT level, whereas addition of 0.1% OA with 0.2% MCT level decreased the ADG of weaning pigs. Treatment LMO showed greater BW for 5 week and ADG for 3 to 5 weeks and overall period than CON treatment. Supplementing 0.2% MCT decreased ADG and G:F ratio for 0 to 2 weeks (MCT, p = 0.04, p = 0.01). Furthermore, 0.2% MCT decreased ADG of pigs for 3 to 5 weeks and overall period (MCT, p = 0.01, p = 0.01).

In the previous study of Kuang et al [3], feed intake had been highly increased by supplementation of 0.3% MCT and OA in 21-day-old weaning pigs for 1 week and 2 weeks. Additionally, BW, ADG, and the G:F ratio were increased by supplementation of MCT with OA during 0 to 1 week. The reason for the good growth performance in the treatment group given MCT with OA additions was due to the effects of MCT as well as the effects of OA in increasing the feed intake and growth rate.

Most previous studies have reported that the supplemental effect of MCT on the growth performance of weaning pigs was positive during phase 1. Loh et al [18] reported that all treatment groups given 5 mL MCT had higher ADG compared to the control group during phase 1 [18]. Similarly, Dierick et al [19] observed that two treatments containing 2.5% MCT showed the highest ADG during 0 to 14 days and had a more than 10% higher ADG and a 3% better feed conversion compared to the control.

The improvement of ADG and feed efficiency of weaning pigs fed the diet with MCT were due to the specific physiological and biological characteristics of MCT. Fatty acids are usually divided into short-chain triglycerides (SCT), MCT, and long-chain triglycerides (LCT) according to their hydrocarbon chain length. LCT comprise of fatty acid chains ranging between 13–22 carbons in length. Compared with LCT, MCT are smaller in molecular weight, water soluble, have lower smoke point and no essential fatty acids. Also, MCT digestion is rapid and simple. MCT do not stimulate cholecystokinin and pancreatic enzymes from the pancreas, and they are directly absorbed into portal circulation [20]. Several effects of these complex MCT would contribute to better absorption capacity of the intestinal tract and lead to a better growth performance [18]. Furthermore, unlike LCT, MCT do not require carnitine. In the intracellular space, LCT bind to carnitine and are transported to the mitochondria for subsequent β-oxidation while MCT do not rely on the carnitine acyltransferase system for transport into the mitochondria for β-oxidation. This provides MCT with a more rapid metabolism and improves their utilization even in protein-deficient conditions [21]. Therefore, MCT have the advantage that they can be used immediately as an energy source and give positive results when was added to weaning pig diets to meet their energy needs. On the other hands, high dosage of dietary MCT may have negative influence on the growth performance of weaning pigs. In earlier experiments by Allee et al [22], high doses (10%) of MCT lowered piglet performance when compared to tallow, lard, or coconut oil. Also, supplementing 2.1% MCT decreased ADFI of weaning pigs linearly between day 1 and 28 among treatments [23]. The treatment with 0.2% MCT showed lower growth performance than the treatment with 0.1% MCT in this experiment. This might be due to not only fatty acid oxidation, which could increase satiety and reduce feed intake, but also the results of lower villous height. The intestinal morphology data in this experiment showed that the VH responsible for nutrient digestion and absorption was relatively shorter in the high MCT-added treatment than in the low-MCT treatment. However, it is difficult to say that adding a higher content of MCT had a negative effect on growth performance in this experiment. Further studies with higher contents of MCT would be warranted.

Consequently, when 0.2% MCT was added, the growth performance was decreased because of fatty acid oxidation which could increase satiety and reduce feed intake and relatively short villous height which could affect digestion and absorption of nutrients. However, supplementation with 0.1% MCT significantly improved ADG and feed efficiency. Furthermore, supplementing 0.1% MCT with 0.1% OA showed significantly higher results in growth performance compared to the control. Our ADG results for MCT and OA interaction effect cannot be related with intestinal morphology results because no significant difference was found in the MCT and OA interaction effect in intestinal morphology in our experiment. Therefore, additional studies related to combination of MCT and OA should be conducted to explain clearly about the interaction effects of MCT and OA to see the growth performance related to the intestinal morphology.

### Fecal score

The effects of MCT with OA on the fecal score are presented in Table 4. No significant difference was observed during the whole experimental period.

Yen et al [14] have measured the fecal score and the number of diarrhea pigs with and without MCT supplementation (3% MCT), and the fecal scores and the number of diarrheal pigs in the MCT supplemented treatment group were lower than those in the control. Tee et al [24] also noted that diarrhea occurred less often in treatment groups given 2 mL MCT compared to the control. These findings could be supported by another previous study which showed that adding MCT in the feed controlled the antibacterial response of pigs. Petschow et al [25] reported that there were strong *in vitro* and *in vivo* antibacterial effects of medium chain fatty acids in the pig proximal small intestine without growth-promoting antibiotics. These results indicated that pathogenic bacteria, including *Vibrio cholera*, *Salmonella typhimurium*, *Shigella sonnei*, *Haemophilus influenzae*, and *E. coli* were inactivated by medium chain fatty acids or their monoglycerides [25]. Furthermore, MCT may reduce the proliferation of the pathogenic bacteria, which eventually could lead to more energy used for animal growth.

In addition, Tsiloyiannis et al [26] have reported on the effect of OA on pig diarrhea by comparing the effects of OA types, and diarrhea was less frequent in all treatment groups given OA than in the control without OA. This was because piglets had reduced intestinal pH after ingesting OA, which inhibited the growth of bacteria, especially *E. coli* [26]. Also, all treatments including OA (0.2% unprotected OA, 0.1% protected OA, 0.2% protected OA) had lower fecal score compared to the control in weaning pigs during whole experimental periods [27]. This experiment like Tsiloyiannis et al [26] showed that OA could prevent or reduce diarrhea in weaning pigs by inhibiting the growth of pathogenic bacteria in the gastrointestinal tract.

One of the reasons why the results were different with previous research was the MCT or OA content. Yen et al [14] added 3% MCT while our experiment was added 0.1% or 0.2% MCT. Also, 0.1% or 0.2% OA was supplemented while our experiment was supplemented 0% or 0.1% [27]. In conclusion, no significant difference in fecal score was found.

### Blood profiles

The effects of MCT with OA on blood profiles of weaning pigs are presented in Table 5. As a result of the analysis, there was no statistically significant difference in blood profiles during the whole experimental period.

Cortisol is a commonly measured indicator of stress [28]. It was measured in the present study because Han et al [29] reported that MCT could reduce the level of cortisol, to lower levels that seen with antibiotics. However, no significant difference was observed in the current study.

The serum IgG, TNF-α, IL-6, IL-1β, and IL-10 can be considered as an indicator for measurement of immunological responses. No significant differences were found in the current study. However, Kuang et al [3] reported that weaning pigs supplemented with the combinations of 0.3% MCT and OA had lower plasma TNF-α (p<0.05) and higher plasma IgG (p<0.05) concentrations than the weaning pigs fed with the control diets. The increased plasma IgG concentration suggested that the immunity of weaning pigs was improved by the combinations of MCT and OA intake. In addition, there was decreased plasma TNF-α concentration and down-regulated TNF-α expression in the small intestine of the combination of MCT and OA fed weaning pigs in this study. The decrease in feed intake and growth observed in diseased or immunologically challenged pigs was considered to be the result of increased activities of pro-inflammatory cytokines such as TNF-α and IL-1 [30].

The reasons why results of this current experiment were not the same as those of Kuang et al [3], are probably due to the difference in composition and content of the added MCT and OA. In the case of Kuang et al [3], 0.3% MCT and OA were added. MCT was mainly composed with a mixture of lauric acid, myristic acid and capric acid and OA consisted of calcium formate, calcium lactate, citric acid, respectively. On the other hand, 0.1% or 0.2% MCT and 0% and 0.1% OA were added in this experiment. MCT mainly contained caproic acid and caprylic acid and OA mainly contained calcium formate, citric acid, fumaric acid.

The current study did not show any significant differences in blood immune parameters.

### Intestinal morphology

The effects of MCT and OA on small intestinal morphology are presented in Table 6. Supplementing 0.1% MCT increased VH in the duodenum (MCT, p = 0.04) and ileum (MCT, p = 0.03) during phase 1 (0 to 2 weeks). In addition, supplementing 0.1% OA increased duodenal VH (OA, p = 0.01) and the VH:CD ratio (OA, p = 0.04) during phase 2 (3 to 5 weeks).

Czernichow et al [31] have noted that supplementation with 50% MCT + 50% LCT could promote mucosal growth and enhance epithelial cell regeneration to create better-developed intestinal tracts in adult rats compared to 100% LCT treatments. Loh et al [18] have reported that compared with the control, supplementing 5 mL MCT per piglet led to greater duodenal and jejunal VH at 6 h and, 6 and 8 days of age. Also, the VH in the ileum at 8 days in pigs fed 5 mL MCT pet piglet was also significantly higher than that of the control. Thus, MCT not only enhanced nutrient absorption and utilization but also promoted the development of the intestines as a whole to improve growth performance. Consequently, the present study suggests that 0.1% MCT given during phase 1 improved duodenal and ileal VH and strengthened the intestinal tracts, as shown in the previous studies of Czernichow et al [31] and Yen et al [14]. Villi have finer-like protrusions in the epithelial inner layer that help to increase the surface area for digestion and absorption processes [32]. Since the improvement in VH increases absorption and surface area, it directly affects the ability of the intestine to absorb nutrients. Mekbungwan and Yamauchi [33] also found a highly positive relationship between an increased VH and improved growth performance of piglets.

Furthermore, the results of this current experiment showed that VH was increased by the 0.1% OA supplementation during phase 2 and were similar to those of Long et al [34] and Ferrara et al [35]. According to the previous study of Long et al [34], supplementing 0.2% OA1 (a synergistic blend of free and buffered short chain fatty acids [mainly formic acid, acetic acid, and propionic acid]) combined with MCT tended to increase ileal VH (p = 0.07) in weaning pigs. Also, the ratio of VH:CD for the jejunum and ileum was greater (p<0.05) for pigs fed OA1 and OA2 (a synergistic blend of a phenolic compound) compared to the control. Ferrara et al [35] have also shown that VH and CD in the jejunum were slightly higher in the treatment groups fed 1.05% OA and 1.05% OA with 0.3% MCT than in the control in weaning pigs. The authors reported that the supplementation of OA and MCT may result in decreased apoptosis rates at the top of the villi. Therefore, the increase in VH and VH:CD ratio in the duodenum during phase 2 in this current experiment could be the result of 0.1% OA supplementation and effects increasing VH with regards to the results of Long et al [34] and Ferrara et al [35].

Therefore, the supplementation of MCT with OA, as in many previous studies, influenced the morphological changes of the small intestine, such as increased VH. In particular, 0.1% MCT significantly increased duodenal and ileal VH during phase 1, and 0.1% OA significantly increased VH and VH:CD ratio in the duodenum during phase 2 in this current experiment. Overall, supplementation of MCT and OA could improve the absorption and utilization of nutrients to enhance the growth performance.

### Nutrient digestibility

The effects of MCT with OA on nutrient digestibility and nitrogen retention are presented in Table 7. Adding MCT and OA did not affect nutrient digestibility and nitrogen retention.

However, unlike the present results, previous studies have shown that MCT supplementation improved nutrient digestibility. Han et al [29] have reported that compared to antibiotic treatment, supplementing 0.1% MCT significantly improved CP, calcium, phosphorus, and energy digestibility in weaning pigs. Han et al [29] have also reported that lipids affected nutrient digestibility by changing the morphology of the small intestine. In addition, Li et al [23] have reported that supplementation of 1.4% MCT showed significantly higher digestibility of ether extract compared to the control in weaning pigs during day 12 to 14 and day 26 to 28. This was because MCT improved the malabsorption of fat in the case of a contracted absorptive surface or atrophied intestinal villi. Moreover, the treatment supplemented with 1.4% MCT had a better result in the apparent total tract digestibility of DM, which resulted from an integrated improvement in the digestibility of various nutrients.

In conclusion, there are many previous studies where supplementation of MCT resulted in improved nutrient digestibility, but no significant difference was found in this study with MCT and OA additions. The discrepancy may be due to the differences in the composition of the diets, component ratio, and experimental situation. In the case of the Han’s experiment, the experiment was conducted only in supplementation with antibiotics and ZnO not in the control in terms of digestibility. Also, the form of the diet (crumbled vs powdered form) was different which could possibly affect feed intake and digestibility. In the case of the Li’s experiment, the amount of MCT supplemented was 0.7%, 1.4%, and 2.1%, which was different from the 0.1% and 0.2% MCT added in this experiment. These differences could bring different results with this experiment.

## CONCLUSION

The addition of 0.1% MCT in the diets for weaning pigs improved the growth performance of pigs with the increase of duodenal and ileal VH. The addition of 0.1% OA in the diets for weaning pigs increased duodenal VH and VH:CD. However, increasing the level of dietary MCT from 0.1% to 0.2% supplemented with 0.1% OA showed a negative effect on the growth performance of weaning pigs. Therefore, the inclusion of 0.1% MCT and 0.1% OA in the diets for weaning pigs would be beneficial for the growth performance of pigs with an improvement of the gut environment.

## Figures and Tables

**Table 1:** Formula and chemical compositions of the experimental diets during the weaning phase 1 (0 to 2 wk)

Items	Treatment^1)^				

	CON	LM	LMO	HM	HMO
Ingredient (%)					
Expanded corn	45.83	45.73	45.52	45.64	45.43
Soybean meal	37.02	37.04	37.08	37.06	37.09
Soy oil	1.84	1.81	1.88	1.79	1.86
Sweet whey powder	4.00	4.00	4.00	4.00	4.00
Lactose	8.00	8.00	8.00	8.00	8.00
L-lysine-HCl, 78%	0.20	0.20	0.20	0.20	0.20
DL-methionine 80%	0.05	0.04	0.04	0.04	0.04
L-threonine, 99%	0.04	0.04	0.03	0.03	0.04
MDCP	1.43	1.43	1.43	1.43	1.43
Limestone	1.06	1.06	1.06	1.06	1.06
Vitamin mix^2)^	0.10	0.10	0.10	0.10	0.10
Mineral mix^3)^	0.10	0.10	0.10	0.10	0.10
Salt	0.30	0.30	0.30	0.30	0.30
Zinc oxide	0.05	0.05	0.05	0.05	0.05
MCT^4)^	0.00	0.10	0.10	0.20	0.20
Organic acids^4)^	0.00	0.00	0.10	0.00	0.10
Total	100.00	100.00	100.00	100.00	100.00
Chemical composition (calculated value)					
ME (kcal/kg)	3,300.00	3,300.01	3,300.03	3,300.01	3,300.03
SID lysine (%)	1.35	1.35	1.35	1.35	1.35
SID methionine (%)	0.35	0.35	0.35	0.35	0.35
SID threonine (%)	0.86	0.86	0.86	0.86	0.86
Calcium (%)	0.80	0.80	0.80	0.80	0.80
Total phosphorus (%)	0.65	0.65	0.65	0.65	0.65
Chemical composition (analyzed value)					
Moisture (%)	8.90	8.76	8.93	8.80	8.95
Crude protein (%)	19.37	19.77	20.09	19.91	19.73
Crude fat (%)	3.39	3.48	3.73	3.45	3.39
Crude ash (%)	6.08	6.14	6.08	6.07	5.94

MDCP, mono-dicalcium phosphate; MCT, medium chain triglyceride; ME, metabolizable energy; SID, standard ileal digestibility; SBM, soybean meal, OA, organic acids.

1) CON, corn-SBM-based diet; LM, corn-SBM-based diet + MCT 0.1%; HM, corn-SBM-based diet + MCT 0.2%; LMO, corn-SBM-based diet + MCT 0.1% + organic acids 0.1%; HMO, corn-SBM-based diet + MCT 0.2% + organic acids 0.1%.

2) Following quantities of vitamins provided per kg of complete diet: vitamin A, 8,000 IU; vitamin D_3_, 1,800 IU; vitamin E, 80 IU; vitamin K_3_, 2 mg; riboflavin, 7 mg; calcium pantothenic acid, 25 mg; niacin, 27 mg; D-biotin, 200 μg; Vitamin B_12_, 50 μg.

3) Following quantities of minerals provided per kg of complete diet: Fe, 150 mg; Cu, 105 mg; Mn, 51 mg; I, 1 mg; Se, 0.3 mg; Zn, 72 mg.

4) MCT and OA products were provided by E&T company (E&T CO., Ltd, Daejeon, Korea).

**Table 2:** Formula and chemical compositions of the experimental diets during the weaning phase 2 (3 to 5 wk)

Items	Treatment^1)^				

	CON	LM	LMO	HM	HMO
Ingredient (%)					
Expanded corn	57.11	57.02	56.82	56.93	56.73
Soybean meal	32.29	32.31	32.33	32.32	32.35
Soy oil	1.78	1.75	1.83	1.73	1.80
Sweet whey powder	2.00	2.00	2.00	2.00	2.00
Lactose	4.00	4.00	4.00	4.00	4.00
L-lysine-HCl, 78%	0.11	0.11	0.11	0.11	0.11
DL-methionine 80%	0.01	0.01	0.01	0.01	0.01
L-threonine, 99%	0.00	0.00	0.00	0.00	0.00
MDCP	1.22	1.22	1.22	1.22	1.22
Limestone	0.95	0.95	0.95	0.95	0.95
Vitamin mix^2)^	0.10	0.10	0.10	0.10	0.10
Mineral mix^3)^	0.10	0.10	0.10	0.10	0.10
Salt	0.30	0.30	0.30	0.30	0.30
Zinc oxide	0.03	0.03	0.03	0.03	0.03
MCT^4)^	0.00	0.10	0.10	0.20	0.20
Organic acids^4)^	0.00	0.00	0.10	0.00	0.10
Total	100.00	100.00	100.00	100.00	100.00
Chemical composition (calculated value)					
ME (kcal/kg)	3,300.01	3,300.00	3,300.01	3,300.04	3,300.02
SID lysine (%)	1.15	1.15	1.15	1.15	1.15
SID methionine (%)	0.30	0.30	0.30	0.30	0.30
SID threonine (%)	0.74	0.74	0.74	0.74	0.74
Calcium (%)	0.70	0.70	0.70	0.70	0.70
Total phosphorus (%)	0.60	0.60	0.60	0.60	0.60
Chemical composition (analyzed value)					
Moisture (%)	9.02	9.24	9.33	9.22	8.78
Crude protein (%)	18.47	18.37	18.51	18.85	18.67
Crude fat (%)	3.97	3.78	3.53	3.29	3.61
Crude ash (%)	4.99	5.12	5.25	5.17	5.40

MDCP, mono-dicalcium phosphate; MCT, medium chain triglyceride; ME, metabolizable energy; SID, standard ileal digestibility; SBM, soybean meal; OA, organic acids.

1) CON, corn-SBM-based diet; LM, corn-SBM-based diet + MCT 0.1%; HM, corn-SBM-based diet + MCT 0.2%; LMO, corn-SBM-based diet + MCT 0.1% + organic acids 0.1%; HMO, corn-SBM-based diet + MCT 0.2% + organic acids 0.1%.

2) Following quantities of vitamins provided per kg of complete diet: Vitamin A, 8,000 IU; Vitamin D_3_, 1,800 IU; Vitamin E, 80 IU; Vitamin K_3_, 2 mg; riboflavin, 7 mg; calcium pantothenic acid, 25 mg; niacin, 27 mg; D-biotin, 200 μg; Vitamin B_12_, 50 μg.

3) Following quantities of minerals provided per kg of complete diet: Fe, 150 mg; Cu, 105 mg; Mn, 51 mg; I, 1 mg; Se, 0.3 mg; Zn, 72 mg.

4) MCT and OA products were provided by E&T company (E&T CO., Ltd, Daejeon, Korea).

**Table 3:** Effects of medium chain triglycerides and organic acids on growth performance in weaning pigs1)

Items	Treatment^2)^					SEM	p-value			
	
	CON	LM	LMO	HM	HMO		Diet	MCT	OA	MCT×OA
Body weight (kg)										
Initial	7.99	8.00	8.00	8.00	7.99	0.165	-	-	-	-
2 wk	9.60	10.07	10.46	9.58	9.47	0.265	0.14	0.30	0.84	0.72
5 wk	18.12^^bc^^	19.52^^ab^^	20.10^^a^^	18.89^^abc^^	17.75^^c^^	0.382	0.03	0.09	0.73	0.31
Average daily gain (g)										
0 to 2 wk	115	148	176	113	106	10.8	0.13	0.04	0.66	0.47
3 to 5 wk	406^bc^	450^ab^	459^a^	444^ab^	394^c^	8.6	0.04	0.01	0.13	0.04
0 to 5 wk	289^bc^	329^ab^	346^a^	311^abc^	279^c^	8.5	0.03	0.01	0.56	0.10
Average daily feed intake (g)										
0 to 2 wk	231	239	258	228	211	9.9	0.53	0.27	0.95	0.49
3 to 5 wk	685	737	751	693	657	18.7	0.46	0.12	0.79	0.54
0 to 5 wk	444	476	487	448	423	12.3	0.44	0.13	0.81	0.52
Gain:feed ratio										
0 to 2 wk	0.496	0.622	0.681	0.494	0.503	0.0286	0.06	0.01	0.52	0.74
3 to 5 wk	0.592	0.610	0.611	0.640	0.601	0.0114	0.59	0.69	0.47	0.39
0 to 5 wk	0.652	0.692	0.710	0.695	0.659	0.0107	0.34	0.38	0.68	0.24

SEM, standard error of the mean; MCT, medium chain triglyceride; OA, organic acids; SBM, soybean meal.

1) A total 120 weaning pigs was fed from average initial body 8.00±0.694 kg and average final body weight was 18.87±2.160 kg.

2) CON, corn-SBM-based diet; LM, corn-SBM-based diet + MCT 0.1%; HM, corn-SBM-based diet + MCT 0.2%; LMO, corn-SBM-based diet + MCT 0.1% + organic acids 0.1%; HMO, corn-SBM-based diet + MCT 0.2% + organic acids 0.1%.

a–c Means in a same row with different superscript letters significantly differ (p<0.05).

**Table 4:** Effects of medium chain triglycerides and organic acids on the fecal score in weaning pigs

Items	Treatment^1)^					SEM	p-value			
	
	CON	LM	LMO	HM	HMO		Diet	MCT	OA	MCT×OA
Fecal score^2)^										
0 to 2 wk	1.66	1.20	1.15	1.25	1.41	0.075	0.32	0.37	0.76	0.55
3 to 5 wk	1.16	0.75	0.75	0.88	0.91	0.054	0.19	0.31	0.73	0.73
0 to 5 wk	1.41	0.98	0.95	1.04	1.16	0.050	0.10	0.22	0.65	0.50

SEM, standard error of the mean; MCT, medium chain triglyceride; OA, organic acids; SBM, soybean meal.

1) CON, corn-SBM based diet; LM, corn-SBM based diet + MCT 0.1%; LMO, corn-SBM based diet + MCT 0.1% + organic acids 0.1%; HM, corn-SBM based diet + MCT 0.2%; HMO, corn-SBM based diet + MCT 0.2% + organic acids 0.1%.

2) Fecal score: 0, normal feces; 1, moist feces; 2, mild diarrhea; 3, watery diarrhea.

**Table 5:** Effects of medium chain triglycerides and organic acids on blood profiles in weaning pigs1)

Items	Treatment^2)^					SEM	p-value			
	
	CON	LM	LMO	HM	HMO		Diet	MCT	OA	MCT×OA
Cortisol (ng/mL)										
Initial	------------------------------------------ 44.56 --------------------------------------									
2 wk	5.48	16.24	4.68	25.36	10.14	3.551	0.15	0.29	0.06	0.78
5 wk	24.33	14.68	10.58	20.93	18.28	2.887	0.17	0.27	0.58	0.90
IgG (g/mL)										
Initial	------------------------------------------ 9.00 ------------------------------------------					-	-	-	-	-
2 wk	10.43	11.73	10.35	11.01	10.68	0.492	0.87	0.85	0.43	0.63
5 wk	15.67	19.04	14.14	15.15	17.39	1.113	0.31	0.88	0.56	0.13
TNF-α (pg/mL)										
Initial	------------------------------------------ 127.89 ---------------------------------------									
2 wk	147.85	137.85	137.78	117.01	124.94	5.114	0.06	0.11	0.70	0.70
5 wk	134.94	118.18	130.34	106.13	110.33	5.792	0.42	0.19	0.49	0.73
IL-6 (pg/mL)										
Initial	------------------------------------------ 317.56 --------------------------------------									
2 wk	320.49	352.14	329.99	407.52	310.32	51.535	0.89	0.88	0.62	0.75
5 wk	311.86	281.42	289.32	293.08	288.34	34.498	0.99	0.94	0.98	0.93
IL-10 (pg/mL)										
Initial	------------------------------------------ 7.94 ------------------------------------------									
2 wk	7.33	6.48	9.95	7.62	6.17	0.661	0.07	0.30	0.42	0.06
5 wk	6.72	7.46	9.37	7.58	8.77	0.879	0.86	0.90	0.42	0.84
IL-1β (pg/mL)										
Initial	------------------------------------------ 23.61 ------------------------------------------									
2 wk	18.29	17.56	27.47	28.59	24.54	2.928	0.15	0.52	0.64	0.28
5 wk	23.28	12.94	19.49	26.99	19.81	3.898	0.28	0.41	0.97	0.43

SEM, standard error of the mean; MCT, medium chain triglyceride; OA, organic acids; ng, nanogram; pg, picogram; IgG, immunoglobulin G; TNF-α, tumor necrosis factor-α; IL, interleukin; SBM, soybean meal.

1) Least squares means of 5 observations per treatment.

2) CON, corn-SBM based diet; LM, corn-SBM based diet + MCT 0.1%; LMO, corn-SBM based diet + MCT 0.1% + organic acids 0.1%; HM, corn-SBM based diet + MCT 0.2%; HMO, corn-SBM based diet + MCT 0.2% + organic acids 0.1%.

**Table 6:** Effects of medium chain triglycerides and organic acids on histomorphology of small intestine in weaning pigs1)

Items	Treatment^2)^					SEM	p-value			
	
	CON	LM	LMO	HM	HMO		Diet	MCT	OA	MCT×OA
2 wk										
Duodenum										
Villus height (μm)	312.67	340.26	341.08	291.83	251.87	14.687	0.33	0.04	0.51	0.49
Crypt depth (μm)	311.29	329.86	350.44	322.61	363.41	13.627	0.90	0.92	0.33	0.74
VH:CD	1.00	1.03	0.97	0.90	0.69	0.062	0.37	0.13	0.29	0.47
Jejunum										
Villus height (μm)	276.31	314.60	329.08	280.93	311.27	12.723	0.75	0.41	0.47	0.79
Crypt depth (μm)	235.07	236.29	245.73	237.33	274.68	14.016	0.84	0.64	0.47	0.66
VH:CD	1.17	1.33	1.33	1.18	1.13	0.085	0.95	0.36	0.97	0.99
Ileum										
Villus height (μm)	231.82	272.76	292.61	234.63	253.37	10.877	0.16	0.03	0.24	0.97
Crypt depth (μm)	231.23	245.27	261.61	223.23	257.69	13.962	0.76	0.61	0.34	0.72
VH:CD	1.00	1.11	1.11	1.05	0.98	0.082	0.89	0.42	0.63	0.74
5 wk										
Duodenum										
Villus height (μm)	273.63	243.07	313.97	272.59	295.06	12.321	0.09	0.72	0.01	0.13
Crypt depth (μm)	386.08	397.83	327.80	417.09	376.71	22.609	0.70	0.49	0.27	0.76
VH:CD	0.70	0.61	0.95	0.65	0.78	0.071	0.11	0.43	0.04	0.17
Jejunum										
Villus height (μm)	229.19	234.02	281.25	242.76	277.82	11.252	0.41	0.91	0.14	0.81
Crypt depth (μm)	291.93	298.49	250.48	242.08	266.93	11.035	0.60	0.36	0.59	0.11
VH:CD	0.78	0.78	1.12	1.00	1.04	0.056	0.25	0.45	0.10	0.24
Ileum										
Villus height (μm)	198.17	255.16	262.81	255.28	255.85	21.163	0.69	0.92	0.91	0.92
Crypt depth (μm)	215.74	242.83	246.05	242.16	230.89	15.841	0.97	0.83	0.91	0.84
VH:CD	0.91	1.05	1.06	1.05	1.10	0.092	0.96	0.94	0.82	0.77

SEM, standard error of the mean; MCT, medium chain triglyceride; OA, organic acids; VH:CD, villus height to crypt depth ratio; SBM, soybean meal.

1) Least squares means for three pigs per treatment.

2) CON, corn-SBM based diet; LM, corn-SBM based diet + MCT 0.1%; LMO, corn-SBM based diet + MCT 0.1% + organic acids 0.1%; HM, corn-SBM based diet + MCT 0.2%; HMO, corn-SBM based diet + MCT 0.2% + organic acids 0.1%.

**Table 7:** Effects of medium chain triglycerides and organic acids on nutrient digestibility in weaning pigs1)

Items	Treatment^2)^					SEM	p-value			
	
	CON	LM	LMO	HM	HMO		Diet	MCT	OA	MCT×OA
Nutrient digestibility (%)										
Dry matter	96.20	96.82	95.78	96.29	95.68	0.417	0.91	0.74	0.40	0.82
Crude protein	95.70	96.39	94.57	95.91	95.06	0.538	0.77	0.99	0.25	0.66
Crude fat	85.18	86.66	83.66	85.00	83.33	1.597	0.96	0.78	0.53	0.85
Crude ash	90.32	92.00	89.00	90.00	89.67	1.071	0.93	0.78	0.50	0.59
N-retention (g/d)										
N-intake	7.05	6.98	6.45	6.91	7.17	2.557	-	-	-	-
N-feces	2.14	1.76	2.63	1.96	2.54	0.258	0.76	0.92	0.21	0.79
N-urine	3.03	2.52	3.78	2.83	3.54	0.362	0.74	0.96	0.23	0.72
N-retention^3)^	1.88	2.70	0.04	2.12	1.09	0.633	0.68	0.65	0.72	0.20

SEM, standard error of the mean; MCT, medium chain triglyceride; OA, organic acids; SBM, soybean meal.

1) A total of 15 barrows (initial body weight, 12.48±0.37 kg) were used.

2) CON, corn-SBM based diet; LM, corn-SBM based diet + MCT 0.1%; LMO, corn-SBM based diet + MCT 0.1% + organic acids 0.1%; HM, corn-SBM based diet + MCT 0.2%; HMO, corn-SBM based diet + MCT 0.2% + organic acids 0.1%.

3) N retention = N intake (g) − fecal N (g) − urinary N (g).
